# Policy maker and provider knowledge and attitudes regarding the provision of emergency contraceptive pills within Lao PDR

**DOI:** 10.1186/1472-6963-10-212

**Published:** 2010-07-20

**Authors:** Vanphanom Sychareun, Keokedthong Phongsavan, Visanou Hansana, Alongkone Phengsavanh

**Affiliations:** 1 University of Health Sciences, Faculty of Postgraduate Studies, Ministry of Health, Vientiane, Lao PDR; 2 Sethathirath Hospital, Ministry of Health, Vientiane, Lao PDR

## Abstract

**Background:**

The Ministry of Health (MOH) launched the National Reproductive Health Policy in 2005, which included recommendations regarding the use of emergency contraceptive pills (ECP). However, ECP have not yet been introduced officially in the public sector of the Lao PDR. Thus, their availability is limited. Understanding the knowledge of ECP and attitudes about their provision, barriers to use, and availability among health providers and policy makers is essential to successfully incorporate ECP into reproductive health services.

**Methods:**

Qualitative research methods using in-depth interviews were employed to collect data from policy makers and health providers (auxiliary medical staff, nurses, and medical doctors). Altogether, 10 policy makers, 22 public providers, and 10 providers at private clinics were interviewed. Content analysis was applied to analyze the transcribed data.

**Results:**

The majority of policy makers and health care providers had heard about ECP and supported their introduction in the public sector. However, their knowledge was poor, many expressed inconsistent attitudes, and their ability to meet the demand of potential users is limited.

**Conclusions:**

There is a need to train health providers and policy makers on emergency contraception and improve their knowledge about ECP, especially regarding the correct timing of use and the availability of methods. In addition, the general public must be informed of the attributes, side effects, and availability of ECP, and policy makers must facilitate the approval of ECP by the Lao Food and Drug Administration. These interventions could lead to increased access to and demand for ECP.

## Background

The Lao PDR is a low income country with an estimated population of only 5.6 million. The country is divided into 17 provinces and is ethnically and linguistically diverse, and geographically widespread (49 ethnic groups and 20 inhabitants per square km). The country's fertility rate is high (4.07), ranging from 2.04 for urban women to 4.74 for rural women. The maternal mortality rate is also high at 405/100,000 live births. The life expectancy at birth is 63 and the under-five mortality rate is 68 per 1,000 live births. The contraceptive prevalence rate is 38.4% (35% using modern methods and 3.4% using traditional methods). The most popular method currently used for married women is the oral contraceptive pill (16%), followed by injectable contraceptives (10.6%) [1,2].

To help alleviate the unmet need for contraception (the average desired family size was 4.2 for the period 1990-1995), the Ministry of Health (MOH) launched an official Birth Spacing Program with support of the UNFPA in 1993 and began its nationwide implementation in 1996. Government partners included family planning and selected reproductive health service workers who were provided with training on how to disseminate reproductive health and family planning information at the community level [3]. This program was the responsibility of the Mother and Child Health Center (MCH) under the name "Reproductive Health Project". Despite its implementation, only a few methods of contraception are routinely offered in hospitals and MCH clinics at the provincial and district level, including IUD insertion, sterilization (at provincial hospitals only), depo medroxy-progesterone acetate (DMPA), condoms and oral contraceptives (OC). Oral contraceptives and condoms are also available at the village level through mass organizations and from village health volunteers or committee leaders who have received basic training. Condoms and oral contraceptive pills are provided free in the public health care sector. Some other forms of contraception such as vasectomy are discussed by public sector providers, but not commonly used by the population because of low levels of acceptability. Other methods such as implants, female condoms, diaphragms, and emergency contraceptive pills (ECP) have not yet been implemented in the public sector and but are available in some private clinics.

Recently, the MOH (2005) [3] endorsed a National Reproductive Health Policy which included emergency contraceptive pills (ECP) as a family planning component. The policy aimed to promote information about emergency contraception and to ensure the availability of the method at specified government health facilities. However, there are currently no suppliers of ECP within the country, and these drugs are not officially registered by the Food and Drug Administration (FDA) which is a prerequisite for the drug to be officially distributed in the public sector. At present, no pharmaceutical company in Lao PDR is marketing emergency contraceptives. This may be due to poor levels of knowledge regarding emergency contraceptives among people in the medical community and lack motivation for the required effort of getting the drugs registered. The next step needed to be taken for ECPs drugs to get registered is to identify partners to register the ECP products and conduct advocacy with the relevant authorities. However, there are some dedicated emergency contraceptive products sold at some private pharmacies and clinics because they are brought into the country illegally. Some providers prescribe women a higher dosage of the regular combined oral contraceptive pills, which can be taken as emergency contraception. However, this is not a very common practice because the clients do not understand these hormonal pills and many providers know very little about emergency contraception.

Emergency contraceptives (EC) are used to prevent unwanted pregnancy. There are several methods of emergency contraception, such as IUD insertion or hormonal ECP which could be either of a) combined hormonal contraceptives, b) levonorgestrel only in one or two doses (1.5 mg) and finally c) Ulipristalacelat 30 mg [4]. IUDs can be used as an EC method by inserting the IUD within 5 days of unprotected sex. The mechanism of ECP is mainly through inhibiting ovulation.

The public health care system in Laos consists of three levels: central, provincial, and district. At the central level, the Ministry of Health (MOH) is in charge of the National Institute of Public Health, the University of Health Sciences, the Central Pharmaceutical Enterprise, the central hospitals; and nine centers, including the Maternal and Child Health Center (MCH). The MCH centers' primary responsibilities include formulating maternal and child health policies and programs, and coordinating the nationwide provision of maternal and child health services. This includes prioritizing interventions; monitoring progress towards targets; and coordinating national, NGO and donor activities in maternal and child health [5]. At the provincial level, services are coordinated by the provincial health office and include service provision through provincial hospitals (45-240 beds) and supervising/supporting district health offices. The provincial health system is mirrored at the district level, where the district health office oversees services provided at the district hospitals and supports service delivery at the health post or dispensary level. District hospitals have between 15-25 beds and provide care for a population of about 300,000 people. In addition, there are health volunteers in each village who focus on health education, communication, hygiene, and disease prevention [5].

In addition to public health facilities, there are more than 900 private clinics in the country, most of which are located in the capital, Vientiane, and almost 2,000 registered pharmacies, located mostly in urban areas in close proximity to district or provincial hospitals. The private clinics and pharmacies play an important role in providing accessible sexual reproductive health services in urban areas and help improve quality of health care through their resources, expertise, and infrastructure [5].

In the Lao PDR, abortion is illegal and highly restricted; the vast majority of procedures are clandestine and a high proportion is unsafe. Legal abortion is permitted only to save the life of pregnant women; however, there are no clear criteria established regarding indications for termination of pregnancy and there is very limited data on the actual use of abortion. Clandestine abortions are commonly performed under unsanitary conditions by unskilled practitioners using dangerous techniques [5].

Service providers and other health care personnel can play an important role in ensuring the availability and use of EC. Providers' attitudes may also play an important role in introducing EC. However, there are a number of potential barriers to providers introducing EC in any country, including Laos. The first is the erroneous perception that the method is abortifacient. A second barrier pertains to conservative attitudes towards sexuality. Physicians may be reluctant to offer EC in Laos, as in many other developing countries. A third obstacle is continued ignorance of the available methods among a large proportion of the health care providers [6]. A final barrier in Laos is legal, because the drug has not yet been registered by the Lao FDA.

This study aims to explore the knowledge of ECP and attitudes about provision of ECP among policy makers and health providers in both the public and private sector. Information gained from this study will inform policy makers about the necessary interventions to increase access to and demand for ECP, including: (1) increasing knowledge of ECP among health providers and policy makers, (2) increasing knowledge of ECP among the public, and (3) encouraging approval of ECP by the Lao Food and Drug Administration.

## Methods

### Study setting

The study was carried out in Vientiane, the capital of the Lao PDR, between March and June of 2007. Vientiane has a population of 700,000 and has the highest population density in the country. Compared with other parts of the country, the capital city has both higher levels of education and income, and is the center of culture, commerce and administration in Laos. There are 108 private clinics in Vientiane, of which 82 provide sexual reproductive health services. The prevalence of contraceptive use in Vientiane is 61 percent [7]. A rapidly changing economy has an impact upon the sexuality of young people: high levels of internal migration place many young people far away from their villages and cultural norms, with an increased risk of unprotected sexual relations.

### Study participants

The target groups of this study were policy makers, health providers, and staff of public and private clinics. Policy makers were selected from the Ministry of Health, and included the director of the Preventive and Curative Department, the director of the FDA, the director of the Maternal and Child Health Center (MCH), and directors from six central hospitals. Public health providers were medical doctors and nurses working in family planning services, including obstetric-gynaecology wards in the government sectors. Private providers were chosen from those who provide Sexual Reproductive Health (SRH) services. The participants were selected to reflect the public and private sectors as well as SRH professional backgrounds.

### Data Collection

The first author (VS) initially contacted the key informants through colleagues in the Ministry of Health, central hospitals, and private clinics. In-depth interviews were carried out face-to-face with the key informants - 10 policy makers, 22 public providers (12 medical doctors and 10 nurses), and 10 health providers at private clinics. Participants were interviewed in private offices at the Ministry of Health, hospitals, and private clinics by the trained interviewers. The interviewers were from the Faculty of Postgraduate Studies and Research who had medical backgrounds. The objectives of the in-depth interviews were to assess the perception and attitudes towards ECP and to understand the beliefs and experiences in communicating with clients. Guidelines for the in-depth interviews were prepared both in English and Lao (Appendix 1), and the information obtained was fully transcribed from tape recordings for analysis.

The material from the interviews was organized and coded based on a content analysis. The latent analysis is denoted as interpretation of underlying meaning of the text and deals with the relationship aspect, while the manifest deals with the visible and obvious components [8]. The manifest analysis was used in this study. The texts from transcription of the in-depth interviews were read several times to comprehend the material in its entirety. The meaningful units were condensed to shorten the text without losing the message. Next, the condensed meanings were labeled with an open code representing content; then, open codes were compared to investigate differences and similarities, and create categories. Finally, the categories were compared to identify sub-themes and themes.

The major themes were knowledge of ECP and attitudes related to the provision of ECP. Table 1 gives an example of the analysis process. The first interview was coded together by four members of the study team to develop mutually agreed upon definitions for each code and to establish examples of each code; codes were then reviewed and revised. Each interview was then coded separately by two members of the study team. After coding, the two team members met to discuss the results; again, any disagreements in coding were resolved by consensus. Categories and themes were arrived at by consensus between the four authors.

**Table 1:** Summary of themes, categories and codes form analysis

Codes	Categories	Themes
Heard of ECP	Understanding of ECP	Limited knowledge, confusion and misconceptions of ECP
Lack of knowledge of mechanism		
Lack of knowledge of dosing		
Lack of knowledge of timing		
Lack of knowledge of side effects		
Confusion the different names of ECP		

Training	Source of information	
Media		
Study tours		
Maternal & Child Health Center		
Colleagues		
Prescription		

Agree to provide ECP versus disagree	Access to ECP services	Ambivalent atttitudes, hesitations towards increased access to ECP
Back up method		

Young people	Target population	
Rape victim		
Married couples with occasional sexual intercourse		
Everybody		

Health care provider	Distribution channels	
Media		
Maternal and Child		
Obstetric-gynaecological wards		
FDA		

Positive effect	Effect of Availability of ECP	
ECP encourage unsafe sexual behaviors		
Negative effect		
Outweigh of positive effect than negative effect		

No registered drugs	Lack of information	Barriers of use ECP
Clandestine drugs		
No drug supplier		
Lack of awareness of policy		

Shy and embarrassment	Cultural values	
Negative attitudes towards premarital sex and abortion		

Ethical clearance was obtained from the National Ethical Review Board, Ministry of Health, and the Ethical Review Board at the University of Health Sciences. Verbal informed consent was obtained from each participant. All information gained from the study participants was confidential and participants could withdraw their participation anytime during the interview. Pseudonymous were used to ensure anonymity.

## Results

The majority of key informants were male (7 males in 10 policy makers, 13 in 22 public providers; 6 in 10 private clinics). Among the policy makers, three were from the MOH, four were from central hospitals, one was from the Mother and Child Health Center, one was from the military hospital, and one was from the police hospital. Policy makers were the directors of the departments of the MOH and the hospitals. Ten of public providers were medical doctors and twelve were nurses who worked in the gynaecology-obstetric wards and mother and child units that provided sexual reproductive health services. The ten private care providers worked in obstetrics-gynaecology and family planning programs in the public sector and operated their clinics after working hours in the government sector. Nearly two-thirds of key informants had at least nine years of working experiences.

### Limited knowledge, confusion and misconceptions of ECP

Most public health care providers and private clinicians were familiar with the term 'Emergency Contraception Pills' but lacked detailed technical knowledge. Some could cite examples of emergency pills.

"I heard about ECP from our staff, but I do not know the mechanism of action in detail." (Female public health provider, 55 years old)

"I heard about ECP before from my colleague who opened a private clinic - brands such as Postinor, Madonna - and that these drugs should be taken within 12 hours after having intercourse and that there are some effects on hormones that affect the mucous membrane of the uterus." (Male private health provider, 37 years old)

There were some misconceptions and confusion about the different brand names of ECP and the corresponding time that each drug should be taken. There was also confusion about the purpose of each drug. Many providers stated that 'ECP', 'post-coital pills' and 'after morning pills' are different in terms of frequency, dosage, effectiveness, and composition. As two key informants mentioned:

"The terms 'ECP', 'post coital,' and 'morningafter pills' are the same, but the difference is the time that patients taken the medicine. The term 'morning after pills' means that pills cannot be taken until the morning and 'post coital' indicates that they should be used immediately after sex." (Male private provider, 40 years old)

*"'ECP' should be taken about 5 hours after having sexual intercourse; while 'morning pills' should be taken in the morning and their effect is slow." (Female public provider, 45 years old*)

Moreover, the interval between the repeated doses reported by respondents varied from six hours to 12 hours and the time limit for starting the regimen varied from one hour to 72 hours following unprotected sex.

"I think that the first pill of Postinor is taken about 6 hours after having sexual intercourse." (Female public provider, 40 years old)

Among those who knew about ECP, the knowledge they reported generally concerned the dosing schedule, timing, side effects, and mechanism(s) of action, but the provider's supposed "knowledge" was often not accurate. Many erroneously believed that repeated use posed health risks such as breast and ovarian cancer. Few public and private clinics correctly identified nausea and vomiting as possible side effects, and few of them knew that the method worked by inhibiting ovulation.

Most respondents obtained their information from training courses, colleagues, and private providers. Other sources of information were journals, internet pages, formal training and visits to Maternal and Child Health Centers (MCH) in Thailand. The majority of health care providers had never prescribed ECP because their clients did not demand it from them and there was no supply.

"I learned about ECP when I was trained in an obstetric-gynaecology residency program. After I finished the residency program, I did not prescribe ECP to clients because I was never asked to do so." (Female public health provider, 35 years old)

"No doctors prescribe it in the hospital, so midwives do not know it well." (Nurse, 35 years old)

### Ambivalent attitudes and hesitation regarding increased access to ECP

#### Access

The attitudes of health providers towards ECP are ambivalent. The majority of both policy makers and providers had positive attitudes towards ECP and agreed that ECP should be introduced into the public sector. However, some of them expressed conservative attitudes towards ECP.

"I do not agree with introducing ECP in the public sector because it will encourage young people to have more casual sex. So we need to tell the potential users that ECP could be used to prevent unwanted pregnancy, but not prevent STIs." (Male public health provider, 36 years old)

These providers will advise potential users that ECP are an emergency choice only, that they should be used in an emergency situation or as a back up method to prevent an unwanted pregnancy, and that the drugs should not be used regularly. However, they will explain how to use the drug and inform the user about side effects. As a key informant explained:

"Emergency contraception needs to be reserved for emergency situations and it should not be used as a substitute for regular contraception." (Male public health provider, 30 years old)

#### Target population

Some health providers feel a need to limit the use of these drugs among young people because of the frequency of unsafe and unplanned sex within this group. Some health providers want to limit the use of the drugs only for rape victims. Many believe that unmarried youth should not use ECP. If ECP are too convenient to access, youth would forget to use condoms. Promiscuity, premarital sex, and the incidence of STIs would then increase. Other providers would like to restrict the use of the drugs to married couples; only a few providers felt that everybody should have access to the use of ECP.

"Emergency contraception can be used when one partner works away from home and the couple does not live together, or for people who only occasionally have sex." (Male private provider, 50 years old)

#### Distribution channels

The majority of policy makers and health providers thought ECP were an essential component of contraceptive services but preferred distribution through health care providers only. Maternal and Child Health Center (MCH) and the obstetric-gynaecological wards at public health facilities providing family planning services to women were thought to be the most suitable. ECP could be easily integrated into existing services because these institutions already provide contraceptives.

"The MCH and obstetrics-gynaecology sections should be responsible for introducing ECP into the services of family planning because they are responsible for reproductive health care and family planning services."(Male private provider, 45 years old)

"The MCH center should be responsible for the provision of ECP under supervision of the Department of Health Prevention, Ministry of Health." (Female policy maker, 56 years old)

Some mentioned that pharmacies should not provide ECP. According to this view, the demand for ECP would increase if they were available by this route, causing potential users to rely on ECP over more effective, ongoing methods. It was assumed that such a scenario would encourage more casual sex and the incorrect use of ECP.

*"If pharmacies sell ECP, users will use it at will, similar to the Chinese drug 'Ya Chine' *(an abortificiant drug), *and there will be some negative consequences as a result of widespread use of ECP." (Female public health provider, 35 years old)*

#### Barriers to Use

Most key informants, both policy makers and health care providers, mentioned that ECP are not yet registered through the FDA and are therefore clandestine drugs. There is currently no supplier of ECP to the country. A major barrier to the demand for ECP is lack of information, as ECP is never mentioned in the mass media.

"Potential users don't know the drug because it is not widely used within the country and they do not know where they can get it." (Male policy maker, 50 years old)

Nearly all policy makers and health providers are aware that there is a political barrier from policy makers at the Ministry of Health. The Ministry does not currently allow the legal use of ECP in the Lao PDR, however, some policy makers and providers interviewed in this study were not aware of this policy. A few health providers cited that the barriers to ECP were conservative and strict cultural values and norms towards premarital sex and abortion. Other potential barriers are shyness and embarrassment about purchasing the drugs, especially for young people.

#### Availability

Overall, most interviewees predicted more positive than negative effects from easier access to ECP. One positive effect is to prevent unwanted and unplanned pregnancies, especially in the case of rape. In addition, it was believed that the incidence of induced abortion would be reduced, and complications stemming from induced abortions- such as tubal infections, bleeding, perforation of uterus - would be reduced. These policy makers and providers foresee no adverse effect of the use of ECP on STIs. Because health staff provide health education about STIs, it is unlikely that the availability of ECP will increase casual sex and promiscuity and reduce the likelihood of using condoms for dual protection against HIV and STIs.

In contrast to the optimists described above, some policy makers and health care providers hinted, though to varying degrees, towards the negative effects of the general availability of ECP, mentioning increased promiscuity and STIs. As one key informant said:

"I think that people will have more casual sex and STIs will increase because the clients are more afraid of becoming pregnant than getting STIs." (Male policy maker, 47 years old)

However, few policy makers expect that routine contraceptive use will decrease. Most of the key informants are convinced that the positive effects outweighed the negative effects.

"There would be more positive effects from the availability of ECP than negative effects. For example, the number of unwanted pregnancies would be reduced and the number of newborns without fathers also would be reduced. Still, there could be some negative effects, such as an increase in unplanned sex and STIs." (Male policy maker, 55 years old)

## Discussion

This study indicated that both public and private providers are ignorant about the effect and mechanism of action, side effects, indications, and effectiveness of ECP. This corresponds with results from studies conducted elsewhere indicating that the knowledge of health providers on EC is low [9-14]. Most believe that repeated use poses a health risk. Although the available evidence indicates that repeated use is safe [15,16], this is not acknowledged by health professionals in a wide range of settings [17-19]. Such misperceptions are one reason that some health providers are reluctant to provide the pills on multiple occasions.

Most providers have ambivalent attitudes. They agree that ECP should be introduced in the public sector, but they want to limit its use to married couples. As in other studies carried out elsewhere, providers are less positive about EC use by adolescents [13]. Both public and private providers also expressed concern that increased access to the method could encourage unprotected sexual intercourse or reduce routine contraceptive use. However research in a variety of contexts has shown that expanded access does not increase the rate of unprotected sexual intercourse, nor does it change sexual behaviour [20,21]. Other studies found that advanced provision of ECP does not appear to adversely affect use of regular contraception or encourage repeated use of emergency contraception [22-24] .

Health providers will play an important role in providing health education on ECP and distributing the medication once it is approved by the FDA. However, with the current low levels of knowledge and with ambivalent attitudes, these providers can not be relied upon to give accurate and consistent advice to potential clients. Thus, health providers must be educated on all salient aspects of the drugs, including the dosing schedule, timing, side effects, and mechanism(s) of action. Only with accurate information will policy makers be an asset in public health education and advocacy.

Women experience multiple barriers when attempting to access emergency contraception. The main barriers for potential use include widespread lack of knowledge about ECP among women and providers, lack of availability [25], and shyness and embarrassment to obtain the drugs. In Italy, where people are also poorly informed on the available methods, it was found that this was a major obstacle for the more widespread use of emergency contraception [26]. As a consequence of lack of commercial promotion of ECP, very few women know that emergency contraception is available, effective, and safe [27]. Thus, there is a pressing need for a social marketing campaign to educate the general public about ECP and to update them on the approval process of the drugs.

Emergency contraception is not universally available in Laos because these drugs are not registered officially with the FDA; however, they are available at private drugstores (brands such as Postinor and Madonna). The MOH included ECP in its National Reproductive Health Policy in 2005 and MCH centers will be responsible for the provision of ECP. However, because ECP have not yet been approved by the FDA, policy makers need to advocate for and assist in obtaining approval. Approval would facilitate access to and demand for ECP where women have access to government public health facilities. In addition, providers would be more inclined to provide ECP if they was approved by the FDA [6].

### Methodological Considerations

This study applied a qualitative design to explore the perceptions of policy makers and providers on ECP, using in-depth interviews as the major tool. More focus group discussions or a triangulation of methods might have better highlighted this issue. It should be noted that the pharmacist's and clients' perspectives and experiences were not taken into account, and we acknowledge the need to explore this to capture the full picture of the provision of ECP.

Preconceptions of the interviewers, most of whom had medical backgrounds, may have affected the interpretation of data gained from the interviews. To control for this, group checking with the research team and professional colleagues was done after data collection to assess the trustworthiness of the interpretation of the data.

As for qualitative studies in general, our findings cannot be transferred directly to other settings. However, the study can give valuable insights to other researchers working in similar settings. The key informants were selected based on the diversity of the perspectives they could bring to ECP in the urban areas, but not in the rural areas; furthermore, rural health providers' knowledge and attitudes related to ECP should be considered in additional studies of the issue.

## Conclusion

This is the first study on knowledge and attitudes of ECP within the public and private sectors of the Lao PDR. Awareness about ECP among providers and policy makers is low, and knowledge about ECP among health providers is inadequate. In order to introduce ECP into the public and private sector, there is a need to implement an educational campaign. Educational efforts should focus on the training of health care providers with regards to the available methods and correct timing of use. The standard guidelines for EC use should reflect those of the World Health Organization and other international organizations. The key messages should emphasize effect, indication, side effects, and dosage (two-tablet doses). In addition, to confirm the legal status of ECP, they should be approved by the FDA. At the population level, efforts need to be made to educate the population about ECP. The information should include the nature of side effects, the emergency contraceptive pill regimen, and the mechanism by which emergency contraceptive pills disrupt fertilization. These measures would greatly improve women's access to, demand for, and effective use of ECP. In addition, educational campaigns could seek to teach women how to take regular combined contraceptive pills as EC until dedicated ECP becomes available.

## Competing interests

The authors declare that they have no competing interests.

## Authors' contributions

SV made a substantial contribution to the study design, data collection, analysis and interpretation, and draft manuscript. KP reviewed data analysis results and critically revised the manuscript. AP and VH participated in designing survey instruments and analyzing data. All authors read and approved the final manuscript.

## Appendix 1: Guideline for in-depth interview

1. In your opinion, what are the differences betwen Emergency Contraceptive Pills (ECPs), Post - coital pills and morning after pills?

2. Have you ever studied or heard about ECPs before? If yes, from where? What did you learn or hear? Are there any issues on this regard that you would like to know more? (Probe for indications of the use, side effects...) If you have never learned or heard about ECP, what contents that you would like to learn about? What are the reliable source(s) of this information? Why?

3. Attitude of providers towards ECP:

• What is your opinion about telling potential users about ECP as an emergency choice?

• In case of providing ECP for women as an emergency back -up method, what is your opinion about this idea?

4. ECP services for a wider perspective:

• What is your opinion towards ECP as an emergency choice for women?

• What organizations should be involved for appropriate ECP use? What section in the health care facility is suitable for integrating ECP into the services? Why?

• Who should be the potential ECP providers? Why?

•Effect from the availability of ECP towards casual sex or/and prevention? (probe for negative and positive effect to the user)?

• Information dissemination to the public, what is the critical information that should release to the public. What do we expect public to learn about ECP?

5. Are there any ECP available for women at your organization? If yes, what type of ECP. If no, why are ECP not included in your organization?

• Who should be the potential users?

• How do you feel about women who used ECP? Why can potential users not obtain ECP? What are the barriers? Describe? (Probe for information and communication, culture, values and norms, time and transport, price...)

6. What are the appropriate means of providing information for ECP users to strengthen the health services?

• For service providers what are key messages and format?

•Type of printed materials for ECP users. What should it be?

## Pre-publication history

The pre-publication history for this paper can be accessed here:

http://www.biomedcentral.com/1472-6963/10/212/prepub
